# Environmental factors associated with the distribution of *Loa loa* vectors *Chrysops* spp. in Central and West Africa: seeing the forest for the trees

**DOI:** 10.1186/s13071-019-3327-9

**Published:** 2019-02-06

**Authors:** Xavier Badia-Rius, Hannah Betts, David H. Molyneux, Louise A. Kelly-Hope

**Affiliations:** Department of Tropical Disease Biology, Liverpool School of Tropical Medicine, Liverpool, UK

**Keywords:** *Chrysops silacea*, *Chrysops dimidiata*, *Loa loa*, Loiasis, Africa, Ecology, Climate, Environment, Rainforest, MaxEnt

## Abstract

**Background:**

Loiasis is caused by the filarial parasite *Loa loa*, which is widespread through Central and West Africa and largely confined the tropical equatorial rainforests. The tabanid flies *Chrysops silacea* and *Chrysops dimidiata* are the main vectors driving transmission. This study aimed to better define the spatial distribution and ecological niche of the two vectors to help define spatial-temporal risk and target appropriate, timely intervention strategies for filariasis control and elimination programmes.

**Methods:**

*Chrysops* spp*.* distributions were determined by collating information from the published literature into a database, detailing the year, country, locality, latitude/longitude and species collected. Environmental factors including climate, elevation and tree canopy characteristics were summarised for each vector from data obtained from satellite modelled data or imagery, which were also used to identify areas with overt landcover changes. The presence of each *Chrysops* vector was predicted using a maximum entropy species distribution modelling (MaxEnt) method.

**Results:**

A total of 313 location-specific data points from 59 published articles were identified across seven loiasis endemic countries. Of these, 186 sites were included in the climate and elevation analysis, and due to overt landcover changes, 83 sites included in tree canopy analysis and MaxEnt model. Overall, *C. silacea* and *C. dimidiata* were found to have similar ranges; annual mean temperature (24.6 °C and 24.1 °C, respectively), annual precipitation (1848.6 mm and 1868.8 mm), elevation (368.8 m and 400.6 m), tree canopy cover (61.4% and 66.9%) and tree canopy height (22.4 m and 25.1 m). MaxEnt models found tree canopy coverage was a significant environmental variable for both vectors.

**Conclusions:**

The *Chrysops* spp. database and large-scale environmental analysis provides insights into the spatial and ecological parameters of the *L. loa* vectors driving transmission. These may be used to further delineate loiasis risk, which will be important for implementing filariasis control and elimination programmes in the equatorial rainforest region of Central and West Africa.

**Electronic supplementary material:**

The online version of this article (10.1186/s13071-019-3327-9) contains supplementary material, which is available to authorized users.

## Background

*Loa loa* (Cobbold, 1864) is a filarial nematode that causes infection and disease commonly known as loiasis, or African eye worm [1, 2]. *Loa loa* is mainly transmitted by two tabanid flies of the genus *Chrysops* (Order Diptera: Family Tabanidae): *Chrysops silacea* (Austen) and *C. dimidiata* (Wulp), both rainforest canopy dwellers in Central and West Africa [3, 4]. Loiasis distribution has recently been mapped through large-scale community surveys based on the presence of eye worm, and is endemic in 11 countries [2]. The current distribution overlaps with historical maps, vector distributions [5–7] and the tropical dense and mosaic savanna forest region of Africa [8].

Loiasis is a major impediment for onchocerciasis and lymphatic filariasis (LF) elimination programmes implementing mass drug administration (MDA) with ivermectin, due to the risk of severe adverse events (SAEs) in individuals with high *L. loa* microfilariae (mf) levels [1, 9–12]. Loiasis is not officially classified as a neglected tropical disease (NTD), but there is increasing evidence that it is of public health importance [13]. There is a need to understand the factors driving high *L. loa* transmission as they may help to define spatial-temporal risk and target appropriate, timely intervention strategies. Environmental drivers may be particularly important given that vector-borne disease transmission is influenced by a range of climatic and landscape factors [14, 15].

Loiasis prevalence distributions have been associated with forest cover, landcover, vegetation, rainfall, temperature, elevation, humidity and soil types at different spatial scales using different methods, including multi-country maps and modelling [8, 16–18], comparisons between bio-ecological zones [19], and micro-scale analysis within high-risk area [20]. There is a paucity of information for *Chrysops* vectors; however, a recent review of historical studies highlighted that temperature, rainfall, ground water, tree height, forest coverage and/or intensity of light were identified as important factors driving vector biting and infections rates [3].

Given that *C. silacea* and *C. dimidiata* are the dominant *L. loa* vectors in Central and West Africa, this study aimed to better define the spatial distribution and ecological niche of the two vectors. The recent compilation of *Chrysops* information [3], provided the opportunity to retrospectively collate and geo-reference data, create maps, examine environmental factors and develop models associated with the presence of each vector, using advanced satellite remote sensing data, GIS technologies and the species distribution modelling tools.

## Methods

### Vector data and distribution map

To develop a geo-referenced database of the main *L. loa* vectors, all literature on *C. silacea* and *C. dimidiata* available in Kelly-Hope et al. [3] was reviewed to identify studies with location-specific data. For each study identified, the locations of all villages and/or entomological collection sites were geo-referenced using latitude and longitude coordinates obtained from the article directly and cross-checked with Google Earth Pro (https://www.google.com/earth/). Geo-localisation of the sites without coordinates was carried out with data from Google Maps (https://www.google.com/maps), Google Earth Pro and GeoNames (http://www.geonames.org). Villages or collection sites that were not found or where the location was not precise were excluded from the database, and any repeated sites of the same species were excluded from the environmental analyses (described below). All geo-referenced *Chrysops* spp. were imported into the mapping software QGIS 2.14.20 [21], and a map showing the village and/or entomological collection sites of the two *Chrysops* spp. was created.

### Environmental data and analysis

To examine environmental factors associated with the presence of *C. silacea* and *C. dimidiata*, nine climatic, topographical and forest-related variables, which are considered to affect the development and survival of vectors, were examined. First, long-term climate, and elevation data were obtained from the WorldClim 1.4 - Global Climate Data (http://www.worldclim.org) at 1 km resolution, which used interpolations of observed data, representative of 1960–1990 [22]. The bioclimatic variables available are frequently used for species distributions and related ecological modelling. For this study, annual mean temperature (Bio1), mean temperature of the warmest quarter (Bio10), mean temperature of the coldest quarter (Bio11), annual precipitation (Bio12), precipitation of the wettest quarter (Bio16), precipitation of the driest quarter (Bio17) and elevation data were examined. The temperature measures were measured in degree Celsius (°C), precipitation in millimetres (mm) and elevation in metres (m).

Secondly, as both *Chrysops* spp. are forest canopy dwellers, data on tree canopy height and tree canopy coverage were examined. The tree canopy coverage data, defined as canopy closure for all vegetation taller than 5 metres (m) in height at a 30 m resolution and encoded as percentage (%), were generated for the year 2000, and obtained from the Global Forest Change 2000–2016, version 1.4 (https://earthenginepartners.appspot.com/science-2013-global-forest/download_v1.4.html) [23]. The tree canopy height (m) data were obtained from the Global 1 km Forest Canopy Height modelled data generated in 2005 available from ORNL DAAC 2017 [24].

To account for the historical nature of the *Chrysops* spp. data and identify potential anthropogenic changes to vector habitats such as deforestation and/or urbanization, the levels of tree canopy coverage over time were examined. Satellite images available in Google Earth taken since the 1980s were visually compared at three-time points: 1984, the first image available; 2000, year of the tree canopy cover variable; 2018, current. Villages and/or entomological collection sites that had overt visual changes in forest coverage were excluded from canopy height and canopy coverage analyses.

All geo-referenced *Chrysops* spp. and environmental data were imported into QGIS 2.14.20 [21]. First, at each village and/or entomological site a 3 km buffer using geoprocessing spatial tool was created. Secondly, environmental data within the buffers were extracted, and exported for descriptive and statistical analyses using IBM SPSS Statistics 24. Data were summarised, and comparisons between *C. silacea* and *C. dimidiata* conducted using the Mann-Whitney non-parametric test, with a significance level of *P ≤* 0.05.

### MaxEnt model and probability maps

To predict the presence of *C. silacea* and *C. dimidiata* using environmental data, a maximum entropy species distribution modelling (MaxEnt) method was used [25]. The MaxEnt software (version 3.4.1), is a general-purpose learning method, which uses presence-only (i.e. occurrence) data and environmental variables to help define the distribution of the maximum entropy (i.e. closest to uniform) [25–27].

Data points where tree cover had changed over time, i.e. between 1984–2018, were excluded from the analysis. Environmental data included one main temperature and precipitation variable to account for collinearity between the related sub-variables. Elevation was excluded as it was used as a covariate in the WorldClim data production. To account for the different spatial resolutions between WorldClim (1 km), tree canopy coverage (30 m) data and tree canopy height (1 km), re-sampling of the tree cover variable to 1 × 1 km using a bilinear interpolation was conducted in QGIS 2.14.20 [28, 29]. All layers were converted to ASCII (American Standard Code for Information Interchange) format for MaxEnt model use [26].

Thirty replicates of each model for each *Chrysops* spp. were generated by bootstrapping [29, 30]. To evaluate model performance, the data was randomly split in two parts: 75% of the occurrence data was selected as training data to fit the model and 25% as validation data to evaluate model prediction. The maximum number of background points was set to 10,000 and continuous maps of *Chrysops* spp. suitability were obtained by logistic output, which illustrates an estimated probability of presence in terms of probability values ranging from 0 (unsuitable) to 1 (highest suitability) [30].

Threshold independent area under the curve (AUC) was used to interpret the performance of the model. AUC values range from 0 to 1 where 0.5 represents random prediction. For instance, a value of 0.8 indicates that there is a 0.8 probability that a random selected occurrence point has greater predicted suitability value than a random background point [30]. Jackknife test and variable contribution table assessed the importance of each environmental variable in isolation as well as when it was omitted. Finally, the response curves of how the probability of presence changed along different values of each variable was examined [26].

A spatial distribution map was produced representing the probability of occurrence in form of percentages for both vectors *C. silacea* and *C. dimidiata*. The data were obtained from MaxEnt model results and the maps were created using QGIS 2.14.20 [21].

## Results

### *Chrysops* spp. points distribution

**Fig. 1:**
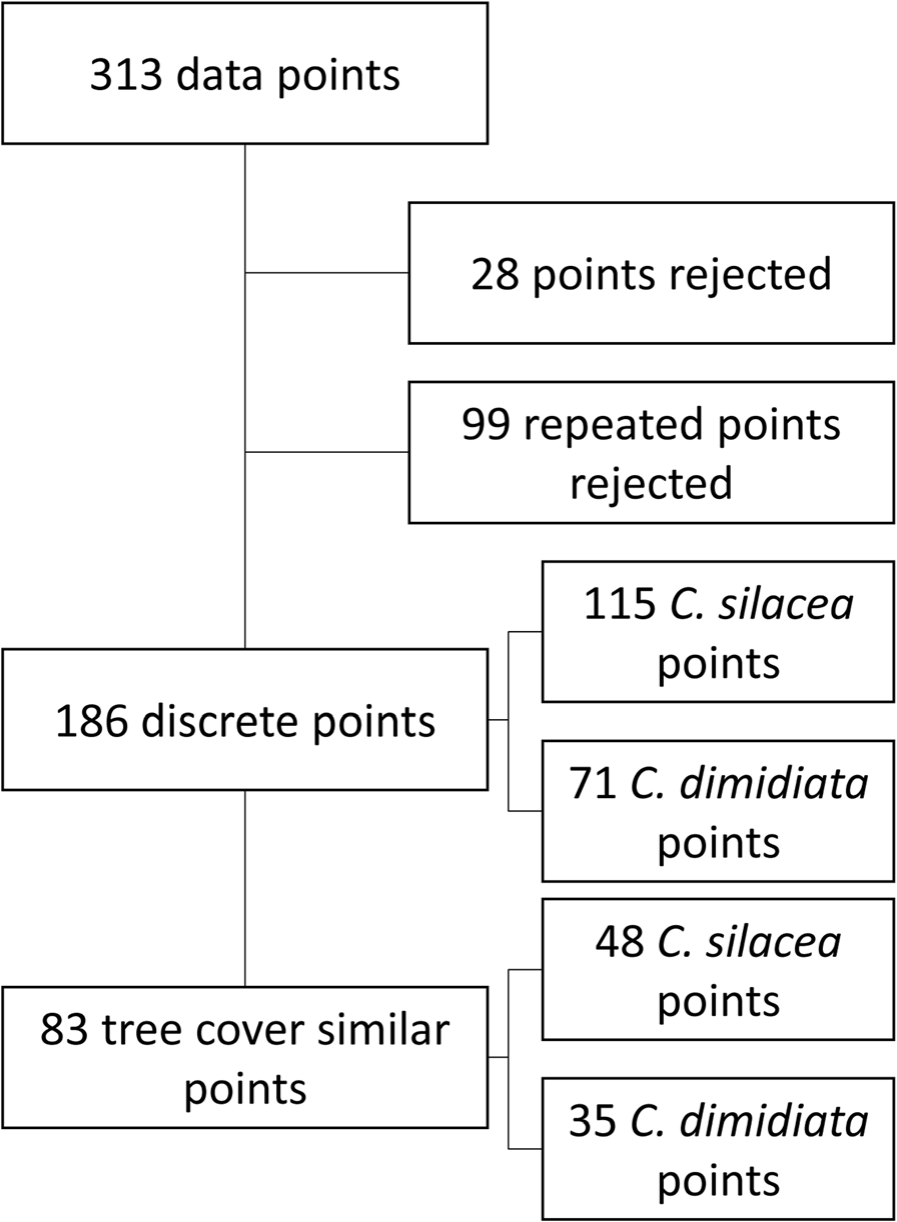
Diagram of methodology used in *Chrysops* data points

**Fig. 2:**
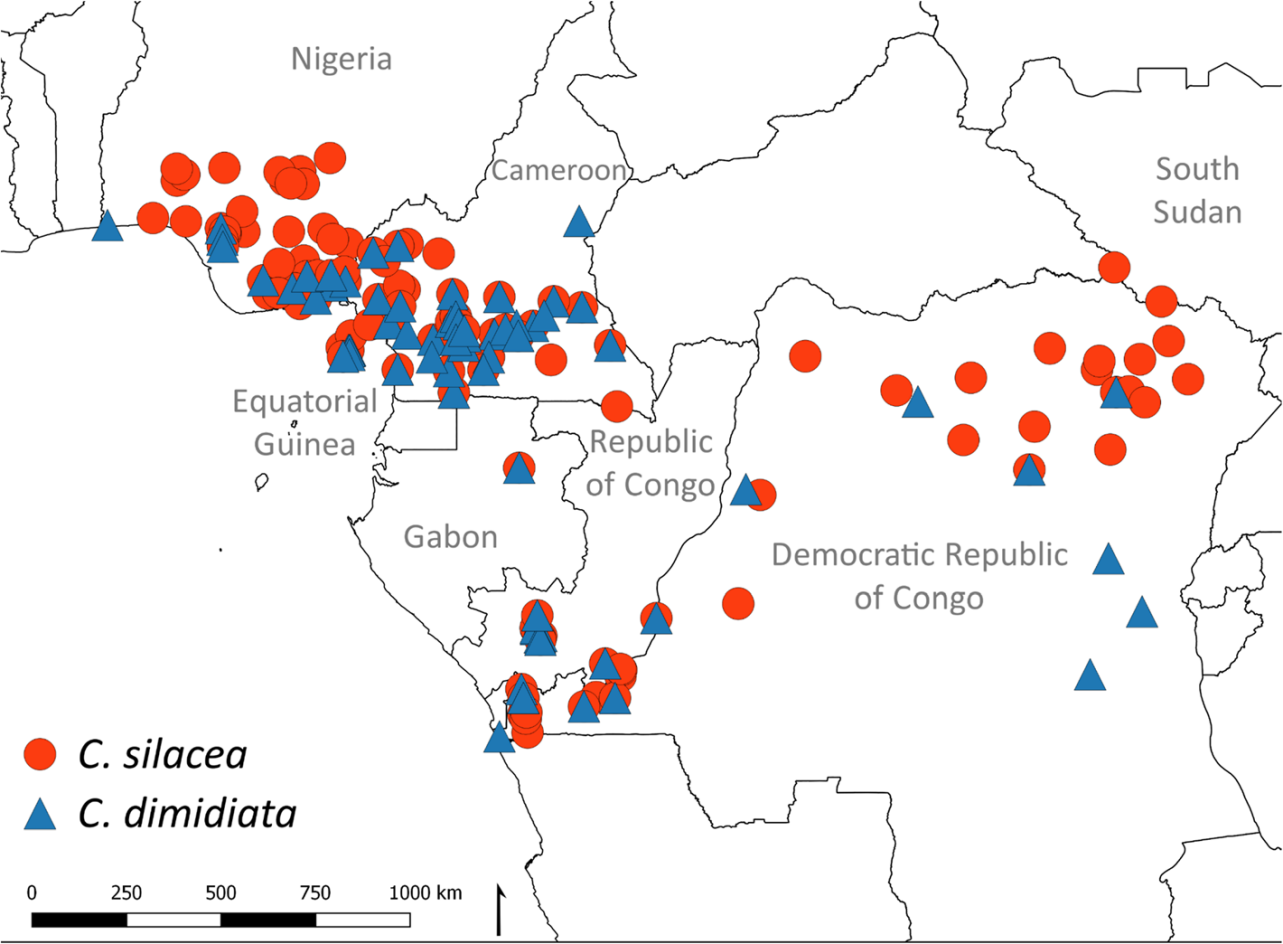
Map of *Chrysops* locations in the Central African countries

In total, 59 articles published between 1912 and 2013 with 313 location-specific data points across Central and West Africa were identified (Fig. 1). Of these, 28 data points were excluded due to lack of a precise location, and 99 data points excluded as they were repeats, i.e. vector collections conducted in the same place at different times. The remaining 186 data points were in Cameroon (*n* = 65), Nigeria (*n* = 48), Equatorial Guinea (*n* = 10), South Sudan (*n* = 2), Democratic Republic of Congo (DRC; *n* = 43), Republic of Congo (Congo; *n* = 16) and Gabon (*n* = 2) (Additional file 1: Table S1). The vector *C. silacea* was recorded in 115 sites and *C. dimidiata* in 71 sites. The distribution of both vectors was similar, and they overlapped in many regions, particularly in Cameroon as shown in Fig. 2.

### Climatic and topographical associations

The summary of the different long-term climate and elevation variables for each species in each country are shown in Table 1. Overall, the environmental parameters for *C. silacea* and *C. dimidiata* were similar and within defined ranges; annual mean temperature (24.6 °C and 24.1 °C, respectively), mean temperature of the warmest quarter (25.7 °C and 25.1 °C), mean temperature of the coldest quarter (23.3 °C and 22.8 °C), annual precipitation (1848.6 mm and 1868.8 mm), precipitation in the wettest quarter (778.3 mm and 789.9 mm), precipitation in the driest quarter (94.5 mm and 100.5 mm) and elevation (368.8 m and 400.6 m). However, *C. silacea* was found to have a significantly higher annual mean temperature, mean temperature of the warmest quarter, mean temperature of the coldest quarter than *C. dimidiata* (*U* = -2.460; *P* = 0.014; *U* = -2.694; *P* = 0.007; and *U* = -1.999; *P* = 0.046, respectively). Overall, there were no significant differences between the vectors for the precipitation and elevation parameters. Interestingly, the country means range for the variables mean temperature of the warmest quarter and coldest quarter was 28 °C and 17 °C, respectively.

**Table 1:** Descriptive statistics of the different environmental variables by vector species and country

Species	Country	*n*	Statistic	Annual mean temperature (°C)	Mean temperature warmest quarter (°C)	Mean temperature coldest quarter (°C)	Annual precipitation (mm)	Precipitation wettest quarter (mm)	Precipitation driest quarter (mm)	Elevation (m)
*C. silacea*	Cameroon	33	Mean	23.4	24.3	22.4	1939.0	854.8	103.0	550.1
			SD	2.7	2.8	2.7	474.3	269.8	38.0	313.7
	DRC	30	Mean	24.9	25.9	23.4	1596.4	591.4	123.2	448.3
			SD	0.8	1.0	1.1	324.3	57.7	108.6	216.6
	Equatorial Guinea	4	Mean	18.0	18.6	17.3	1832.2	772.8	114.0	201.4
			SD	2.7	2.8	2.6	431.4	190.2	25.3	206.5
	Gabon	1	Mean	24.0	24.9	22.4	1646.1	668.6	93.1	493.6
			SD	–	–	–	–	–	–	–
	Nigeria	37	Mean	26.4	27.8	25.0	2063.5	901.0	81.7	127.0
			SD	0.7	0.7	0.8	597.5	253.4	44.5	137.0
	Congo	8	Mean	23.9	25.1	21.8	1559.8	657.2	13.0	428.6
			SD	0.9	0.7	1.3	85.7	53.5	13.5	55.1
	South Sudan	2	Mean	24.7	26.0	23.5	1454.3	597.8	46.1	687.7
			SD	0.3	0.6	0.0	3.4	19.4	2.5	49.8
	Overall	115	Mean	24.6	25.7	23.3	1848.6	778.3	94.5	368.8
			SD	2.4	2.6	2.3	501.0	245.3	69.8	283.1
*C. dimidiata*	Cameroon	32	Mean	23.7	24.6	22.7	1849.5	794.9	103.8	540.1
			SD	2.5	2.6	2.4	435.4	229.1	35.6	275.6
	DRC	13	Mean	24.6	25.6	23.0	1505.2	572.2	123.8	415.0
			SD	0.7	0.9	1.3	359.2	64.8	128.8	230.2
	Equatorial Guinea	6	Mean	21.0	21.6	20.1	2145.8	906.6	127.2	235.1
			SD	3.4	3.5	3.3	385.5	159.5	23.3	150.9
	Gabon	1	Mean	24.0	24.9	22.4	1646.1	668.6	93.1	493.6
			SD	–	–	–	–	–	–	–
	Nigeria	11	Mean	26.5	27.6	25.3	2448.4	1076.2	113.2	39.4
			SD	0.4	0.5	0.3	447.5	165.1	27.4	35.9
	Congo	8	Mean	23.9	25.1	21.8	1559.8	657.2	13.0	428.6
			SD	0.9	0.7	1.3	85.7	53.5	13.5	55.1
	Overall	71	Mean	24.1	25.1	22.8	1868.8	789.9	100.5	400.6
			SD	2.4	2.5	2.3	491.7	234.6	68.1	277.2

*Abbreviation*: *DRC* Democratic Republic of the Congo, *SD* standard deviation

Comparisons between countries found that the *C. silacea* and *C. dimidiata* sites in Nigeria had a higher mean annual temperature (26.4 °C and 26.5 °C, respectively), higher annual precipitation (2063.5 mm and 2448.4 mm) and lower elevation (127.0 m and 39.4 m) than all other countries; however, only a few data points were available for Equatorial Guinea, Gabon and South Sudan (Table 1).

### Tree canopy associations

Examination of satellite images available in Google Earth found that 103 of the 186 sites (55.4%) had overt landcover changes and were excluded from tree canopy analysis. Two examples of sites with minimal landcover changes and two with overt landcover changes are presented in Additional file 2: Figure S1. The exclusion of the sites with overt changes resulted in 48 sites for *C. silacea* and 35 sites for *C. dimidiata* available for analyses and their distribution is shown in Fig. 1.

Overall the mean tree canopy height and canopy coverage for *C. silacea* sites were lower (22.4 m and 61.4%, respectively) than those for *C. dimidiata* sites (25.1 m and 66.9%) (Table 2); however, the differences were not statistically significant.

**Table 2:** Descriptive statistics of canopy height and tree canopy cover variables by species and country

Species	Country	*n*	Statistic	Canopy height (m)	Tree cover (% canopy > 5 m)
*C. silacea*	Cameroon	12	Mean	29.0	65.0
			SD	5.4	13.3
	DRC	14	Mean	20.7	72.3
			SD	10.7	19.2
	Equatorial Guinea	3	Mean	26.0	56.2
			SD	5.0	18.1
	Nigeria	10	Mean	17.3	34.0
			SD	9.8	21.4
	Congo	8	Mean	21.1	74.3
			SD	6.2	21.3
	South Sudan	1	Mean	15.7	51.9
			SD	–	–
	Overall	48	Mean	22.4	61.4
			SD	9.1	23.3
*C. dimidiata*	Cameroon	12	Mean	28.0	62.3
			SD	5.9	12.6
	DRC	7	Mean	20.2	73.2
			SD	12.6	23.1
	Equatorial Guinea	6	Mean	29.4	63.2
			SD	5.9	16.8
	Nigeria	2	Mean	27.0	53.0
			SD	8.1	27.1
	Congo	8	Mean	21.1	74.3
			SD	6.2	21.3
	Overall	35	Mean	25.1	66.9
			SD	8.3	18.5

*Abbreviation*: *DRC* Democratic Republic of the Congo, *SD* standard deviation

Comparisons between countries found that *C. silacea* highest mean canopy height was in Cameroon (29.0 m) and Equatorial Guinea (26.0 m), and the highest canopy coverage was in DRC (72.3%) and Congo (74.3%); however, only a few data points were available for Equatorial Guinea and South Sudan. For the *C. dimidiata* sites, the highest canopy height was found in DRC (28.0 m) and Equatorial Guinea (29.4 m), and the highest canopy coverage in DRC (73.2%) and Congo (74.3%); however, only a few data points were available for Nigeria (Table 2).

### MaxEnt model and probability maps

**Fig. 3:**
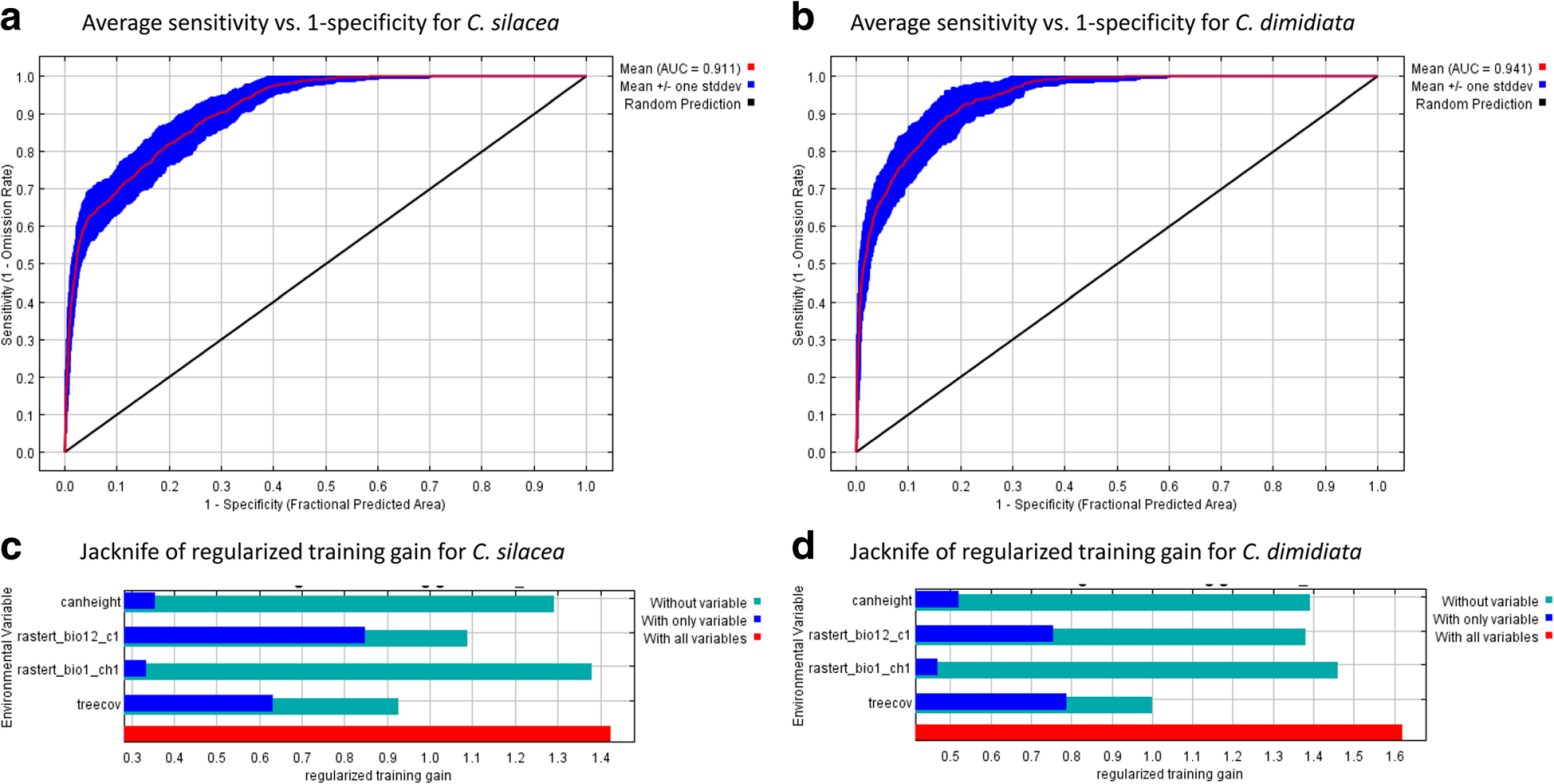
MaxEnt model results plots. **a**, **b** Area under the curve (AUC) plots of both species models. Red line shows the mean of the 30 replicate MaxEnt runs and blue area the mean ± one standard deviation. **c**, **d** Jackknife test of regularized training gain. Dark blue columns show how would be the model gain using each variable in isolation. Light blue columns show how would change the model gain if the variable was excluded. The longest dark blue column turns to be the variable to have the most useful information by itself. The shortest light blue column appears to be the variable which has the most information that is not present in other variables

The MaxEnt model included 83 sites (48 for *C. silacea* and 35 for *C. dimidiata*) and the mean annual temperature, annual precipitation, tree canopy height and tree canopy coverage variables. For *C. silacea*, the model had a mean training AUC of 0.911 ± 0.015 of 30 bootstrapping replications, and for *C. dimidiata,* the model had a mean training AUC of 0.941 ± 0.014, which demonstrated that both models had a robust prediction of the distribution of *Chrysops* vectors. The AUC plot for *C. silacea* and *C. dimidiata* are shown in Fig. 3a and b, respectively. The jackknife test for both species had similar variable contributions. For *C. silacea*, the annual precipitation was the most important variable if considered in isolation. However, if a multivariate model was considered, tree canopy coverage was the variable which when omitted reduced the fit of the model the most (Fig. 3c). For *C. dimidiata*, tree canopy coverage was the most important variable if considered in isolation. Furthermore, it was also the variable which reduced the fit of the model the most when it was omitted in a multivariate model (Fig. 3d). The permutation of the environmental variables in the model indicated that annual precipitation contributed the most for both *C. silacea* and *C. dimidiata* as shown in Table 3*.*

**Table 3:** Permutation importance of the environmental variables in the MaxEnt model

Variable	Permutation importance (%)	
	*C. silacea*	*C. dimidiata*
Annual mean temperature	13.1	35
Annual precipitation	48.8	25.4
Tree cover	23.3	22.9
Canopy height	14.8	16.6

*Note*. Permutation importance depends only on the final MaxEnt model. The contribution for each variable is determined by randomly permuting the values of that variable among the training points (both presence and background) and measuring the resulting decrease in training AUC. Values are normalized to give percentages

**Fig. 4:**
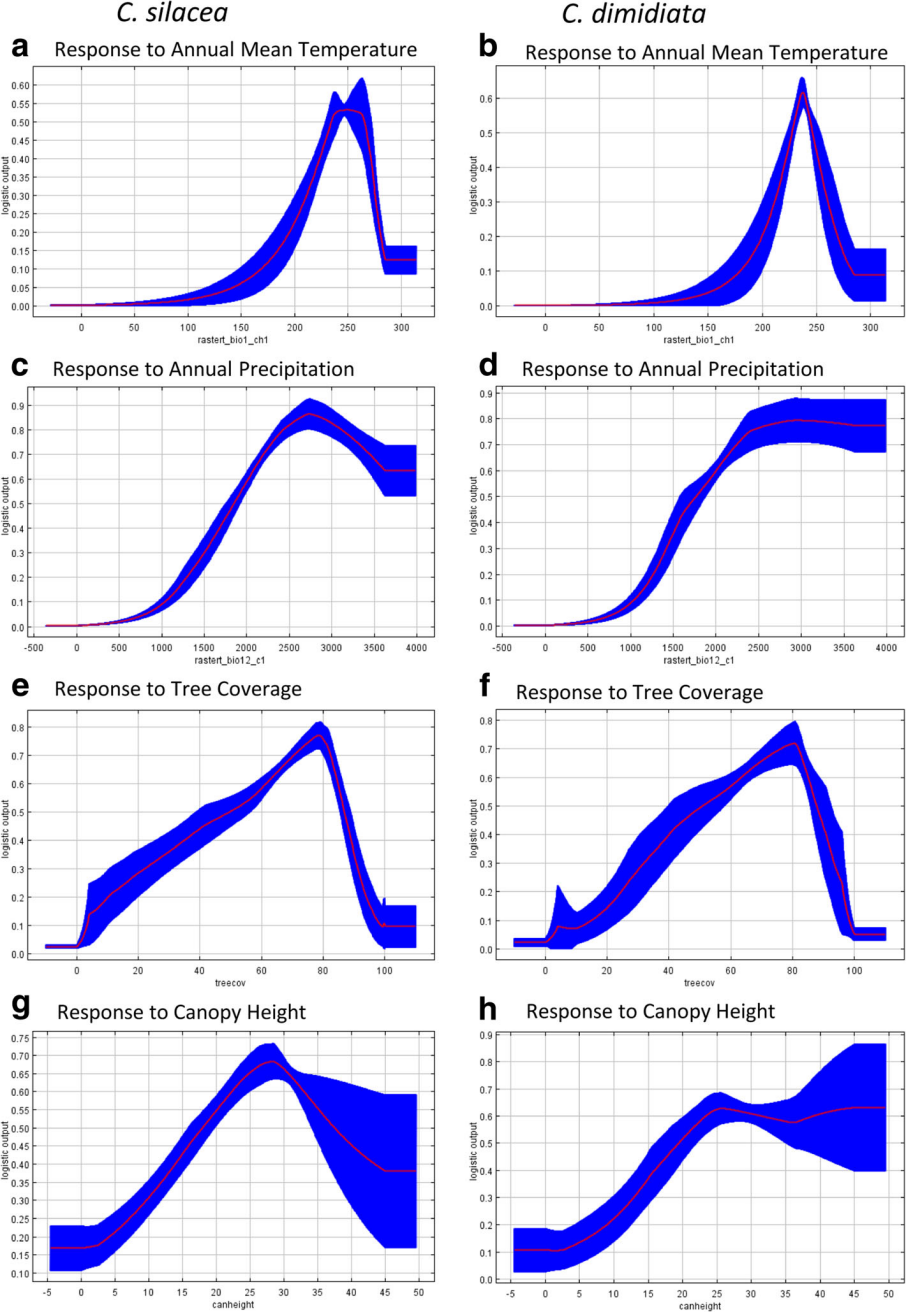
Response curves of environmental variables in the two MaxEnt models for *C. silacea* (**a**, **c**, **e**, **g**) and *C. dimidiata* (**b**, **d**, **f**, **h**). The plots represent a MaxEnt model created using only the corresponding variable. The curves show the mean response of the 30 replicate MaxEnt runs (red line) and the mean ± one standard deviation (blue area)

The response curves highlighted how prediction is affected by each variable. Both species had similar response curve distributions as shown in Fig. 4 a-h. The mean temperature for *C. silacea* indicated a broader temperature niche (Fig. 4a) than for *C. dimidiata* (Fig. 4b). The annual precipitation was similar for both species at 2700 mm; however, the likelihood of *C. silacea* presence was reduced when precipitation was higher than 2700 mm (Fig. 4c), but this reduction was not observed for *C. dimidiata* (Fig. 4d). For both species, tree canopy coverage higher than 80% was not suitable (Fig. 4 e, f). Canopy height response curve was found to have high variation due to its low importance in the prediction (Fig. 4g, h).

**Fig. 5:**
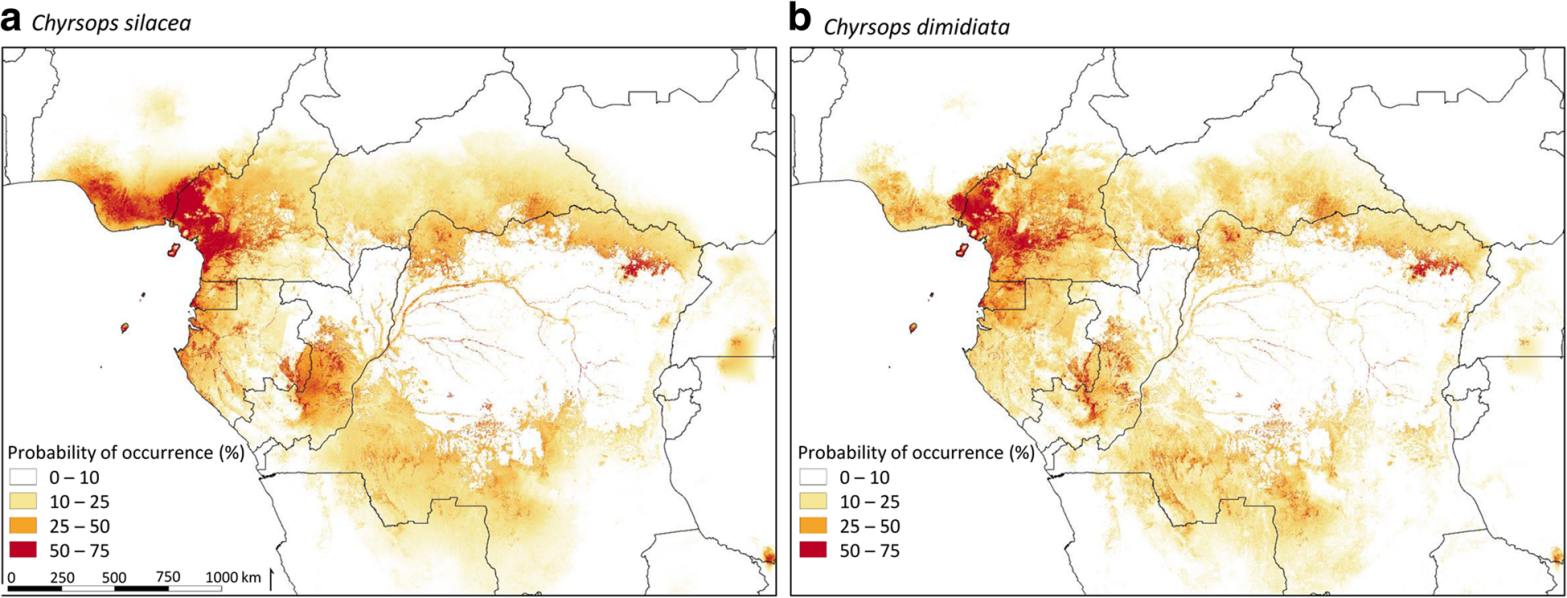
Predicted distribution maps for *C. silacea* (**a**) and *C. dimidiata* (**b**) vectors obtained from MaxEnt model data. Probability of occurrence is depicted in the form of percentages

The predicted distribution of both *Chrysops* vectors from MaxEnt model is shown in Fig. 5a, b. Overall, the distributions were similar; however, *C. silacea* had higher probabilities of occurrence than *C. dimidiata.* The areas with the highest suitability were in southern Nigeria, in southern-central and western Cameroon, in Bioko Island of Equatorial Guinea, all of Gabon, in southern-central Congo, in western and north-eastern DRC, in central CAR and northern Angola.

## Discussion

To our knowledge, this is the first study to collate, geolocate and analyse environmental factors related to the two main vectors responsible for *L. loa* transmission in Central and West Africa. The study highlights the need for more current data on the spatial distribution and ecology of *C. silacea* and *C. dimidiata*, which is fundamental todelineating the risk of filariasis for control and elimination programmes. While there were limitations with using historical vector data, the examination of long-term satellite-derived climate data and landcover changes helped to address these limitations. The results provide several insights into the ecological niche or bio-climate envelope of the two *Chrysops* vectors, and help to define a range of key climatic, topographic and tree canopy parameters using advanced satellite remote sensing data, GIS technologies and the species distribution modelling tools.

Overall, no major differences between the two species were found, apart from temperature where *C. silacea* was found in warmer locations than *C. dimidiata*. Historical studies highlight that temperature is an important factor influencing transmission. For *C. silacea,* vector monthly and/or daily temperatures around 20–28 °C have been shown to be optimal for larvae development, adult density, biting and infection rates [31, 32]. Temperatures lower than 20 °C either in laboratory or very shaded field conditions have shown to delay larvae development in the fly. Noireau et al. [33] reported monthly temperatures, and Crewe & O’Rourke [34] examined hourly temperature fluctuations and found the highest biting activity occurred when temperatures were around 24–27 °C. Interestingly, these finer scale spatial and temporal observations correlate with our large-scale environmental analysis and models, which identify temperature in the mid-20s °C as an important climatic measure.

The level of precipitation in different tropical forest settings may influence the amount of nutrient rich leaves decaying in wet mud available for *Chrysops* larval breeding habitats [3]. Annual precipitation was found to be at least 1500 mm across all countries for both vectors, with the mean rainy season measures (wettest quarter) ranging between 572–1076 mm and mean dry season measures (driest quarter) between 13–127 mm. This study did not examine *Chrysops* temporal conditions; however, several studies have shown that the highest vector biting densities and/or infectivity rates occur during or after the rainy season when ground water and soil moisture may be optimal for breeding [33, 35–37]. Determining the relationship between transmission and precipitation may help to develop a ‘*L. loa* transmission calendar’ highlighting high-risk times [14].

Historical studies also suggest that the extent of forest cover and intensity of light influence transmission [31, 38–40]. In general, *Chrysops* have shown to avoid extreme conditions, including bright sunlight in cleared areas and deep shade in heavily forested areas, with only certain levels of forest and illumination associated with high biting rates. Differences between species have also been found with *C. silacea* being more dominant in cleared forested areas, particularly in villages and their immediate vicinity, and *C. dimidiata* being more closely associated with forested areas [41]. This broadly correlates with our results, which found mean tree canopy coverage was not less than 34% (i.e. cleared areas) or more than 74% (i.e. heavily forested or shaded areas), and specifically that *C. silacea* sites had lower mean proportions of canopy coverage than *C. dimidiata.*

Further, landcover changes may significantly alter transmission potential, especially deforestation as it destroys *Chrysops* spp. habitats. Our retrospective analysis of satellite imagery highlighted that around half of the sites had overt environmental changes over the past few decades, which appeared to be related to population growth and urbanisation. This suggests that the risk of *L. loa* has reduced in these areas, as commercial deforestation, forest clearings caused by population growth with resultant town development and urbanisation, have been associated with a reduction in prevalence [42]. Forest clearings have also been suggested as a potential vector control intervention in certain settings in order to distance the breeding habitats from the human population [5]. This has implications for *L. loa* endemic countries in the Congo Basin region where there is widespread evidence of deforestation and development, which may change how risk is measured and the type of interventions deployed in the future.

Defining spatial-temporal patterns of *Chrysops* spp. and their ecological niches within communities could help to direct interventions, which may include specific vector control measures appropriate for outdoor day-biting vectors, similar to those being developed for other vector-borne diseases [43, 44]. Historical studies highlight effective *Chrysops* defensive control methods such as personal insecticide repellents on the skin or impregnated on clothing to prevent vector biting [3, 5], which may be a practical, cost-effective and scalable intervention for ecologically defined high-risk communities. Aggressive control methods have also been suggested, and indoor residual spraying (IRS) of insecticide to houses or undergrown near breeding sites, or bespoke trapping with or without insecticide, or by using wood fire as an attractant [37, 45], could also be directed to ecologically defined high-risk communities to help reduce the abundance and transmission potential of *L. loa.* This will help filariasis control and elimination programmes overall by reducing the numbers of infective larvae to which humans will be exposed, with the consequence of fewer adult worms producing lower levels of microfilariae, and hence the risk of SAEs.

## Conclusion

The *Chrysops* spp. database and large-scale environmental analysis provides insights into the spatial and ecological parameters of the *L. loa* vectors driving transmission. These may be used to further delineate loiasis risk, which will be important for implementing filariasis control and elimination programmes in the equatorial rainforest region of Central and West Africa.

## Additional files

Additional file 1: **Table S1.** *Chrysops* spp. location database. (XLSX 28 kb)

Additional file 2: **Figure S1.** Satellite images from two sites for the years 1984, 2000 and 2018. **a** Two site examples with similar tree cover. **b** Two site examples with changed tree cover. (DOCX 4054 kb)

## Abbreviations

ASCII: American Standard Code for Information Interchange
AUC: Area under the curve
DRC: Democratic Republic of the Congo
DEC: Diethylcarbamazine
IRS: Indoor residual spraying
LF: Lymphatic filariasis
MaxEnt: Maximum entropy species distribution modelling
mf: Microfilariae
MDA: Mass drug administration
NTD: Neglected tropical diseases
WHO: World Health Organization
RAPLOA: Rapid assessment procedure for loiasis
SAE: Severe adverse events
SDAT: Spatial data access tool
