# Supplementary material for: Environmental factors associated with the distribution of Loa loa vectors Chrysops spp. in Central and West Africa: seeing the forest for the trees
Source: Parasit Vectors. 2019 Feb 6;12:72. doi: 10.1186/s13071-019-3327-9 (PMC6366063; doi:10.1186/s13071-019-3327-9)
Supplement: Supplementary file 2 — Figure S1. Satellite images from two sites for the years 1984, 2000 and 2018. a Two site examples with similar tree cover. b Two site examples with changed tree cover. (DOCX 4054 kb) [file 13071_2019_3327_MOESM2_ESM.docx]

**Additional file 2: Figure S1. Satellite images from two sites for the years 1984, 2000 and 2018**

**a** Two site examples with similar tree cover

1984 2000 2018

**b** Two site examples with changed tree cover

1984 2000 2018

Satellite images were taken from Google Earth Pro version 7.3.1.4505 (2018 Google Inc.) from years 1984 (A, D, G, J), 2000 (B, E, H, K) and 2018 (C, F, I, L). The yellow circle draws a 3km buffer. First two locations were classified as similar tree cover and included Tinto, Cameroon (A, B, C) and Kasese, DRC (D, E, F). The second two locations were classified as changed tree cover. Oshogbo, Nigeria (G, H, I) and Yambio, South Sudan (J, K, L).
